# Analytical Model for the Depth Progress during Laser Micromachining of V-Shaped Grooves

**DOI:** 10.3390/mi13060870

**Published:** 2022-05-31

**Authors:** Daniel Holder, Rudolf Weber, Thomas Graf

**Affiliations:** Institut für Strahlwerkzeuge (IFSW), University of Stuttgart, Pfaffenwaldring 43, 70569 Stuttgart, Germany; rudolf.weber@ifsw.uni-stuttgart.de (R.W.); thomas.graf@ifsw.uni-stuttgart.de (T.G.)

**Keywords:** laser micromachining, grooves, ultrashort laser pulses, analytical model, depth progress

## Abstract

An analytical model is presented that allows predicting the progress and the final depth obtained by laser micromachining of grooves in metals with ultrashort laser pulses. The model assumes that micromachined grooves feature a V-shaped geometry and that the fluence absorbed along the walls is distributed with a linear increase from the edge to the tip of the groove. The depth progress of the processed groove is recursively calculated based on the depth increments induced by successive scans of the laser beam along the groove. The experimental validation confirms the model and its assumptions for micromachining of grooves in a Ti-alloy with femtosecond pulses and different pulse energies, repetition rates, scanning speeds and number of scans.

## 1. Introduction

Laser micromachining of grooves with ultrashort laser pulses is a versatile process that can be applied for various applications, such as cutting through thin metal foils [1], dicing silicon wafers [2], engraving implants made of titanium alloy to enhance osseointegration [3] or engraving cutting tools to reduce the force and friction in mechanical machining of aluminum [4]. In micromachining processes with pulsed lasers, a relative movement between the laser beam and the workpiece is created either by deflecting the laser beam over the processed surface by means of a scanner or by moving the workpiece past the static beam by means of a linear axis. In both cases, multiple scans over the same contour are typically performed to fabricate grooves with a required depth. The dimensions of the micromachined grooves, i.e., the depth and width or their aspect ratio (depth/width), have a major influence on the performance of the respective application: A complete cut through the sample is required in the cutting of thin metal sheets [1]. In open microfluidic systems, a higher flow velocity of liquids is achieved for grooves with a higher aspect ratio [5,6]. Grooves with higher aspect ratios also enhanced the performance of engraved cutting tools in mechanical machining [4].

The laser micromachined grooves with depths of a few tens up to several hundreds of micrometers typically feature a V-shaped geometry in metals [7,8], semiconductors [2,9], dielectrics [10] and polymers [11]. The resulting width of the grooves mainly depends on the diameter of the laser beam, the incident peak fluence and the material-specific ablation threshold [12]. Experimental results revealed the influence of various processing parameters on the resulting depth of a micromachined groove, such as the pulse energy or the irradiated peak fluence, the scanning speed, the number of scans over the surface [2,8,9] and the ablation threshold [12]. The groove’s depth can be increased by multiple scans, whereas high pulse energies [2,8] and low scanning speeds [9] result in a higher increase in the depth for each scan. The progress of the depth of micromachined grooves exhibited a linear correlation with the number of scans for cutting through thin metal foils with a thickness of up to 50 µm [1] and for micromachining of shallow grooves with a depth of up to a few tens of micrometers in semiconductors [9]. A constant deepening rate of laser-processed grooves was achieved up to an aspect ratio (depth/width) of approximately 1.5 [6], which corresponds to the phase of constant depth progress observed in areal micromachining of metals [13] and silicon [14], where the aspect ratio is typically < 1. The depth progress gradually slows down with increasing depth of the groove [6,8,9], reaching a limit when the absorbed fluence is reduced to the value of the ablation threshold everywhere on the walls of the groove [2]. This behavior corresponds to the one found for percussion drilling of microholes, where a decreasing rate of the depth progress was observed with the increasing number of applied pulses, which finally stagnated at the maximum reachable depth [15,16]. It is not energy-efficient to process until this maximum depth is reached since the rate of the depth progress decreases despite the constant applied average power.

The in situ measurement of the current depth during processing has already been demonstrated using optical coherence tomography for percussion drilling of microholes [15,17] and for areal micromachining [18,19]. This approach is also suitable for the production of grooves, as shown in [6] for groove depths up to 500 µm. The prediction of the reachable depth of the grooves or the estimate of the rate of the depth progress during laser micromachining, which would allow for the design of an efficient and productive machining process, has proven to be difficult due to the influence and interplay of various laser parameters, scanning parameters and material properties, including the groove’s depth itself. A numerical model for the calculation of the groove geometry is proposed in [20], but the model does not consider the increased absorptance in V-shaped grooves that is caused by multiple reflections, which were observed in raytracing simulations [21]. 

A simplified analytical model for the prediction of the depth progress in laser machining of V-shaped grooves with ultrashort laser pulses is therefore introduced in the following section. The model was experimentally verified for the case of laser micromachining of grooves with a depth of up to $624_{-38}^{+28}$ µm in the Ti-alloy Ti6Al4V with different pulse energies, repetition rates, scanning speeds and number of scans. 

## 2. Analytical Model for the Prediction of the Depth and Width of Laser Micromachined Grooves

**Figure 1:**
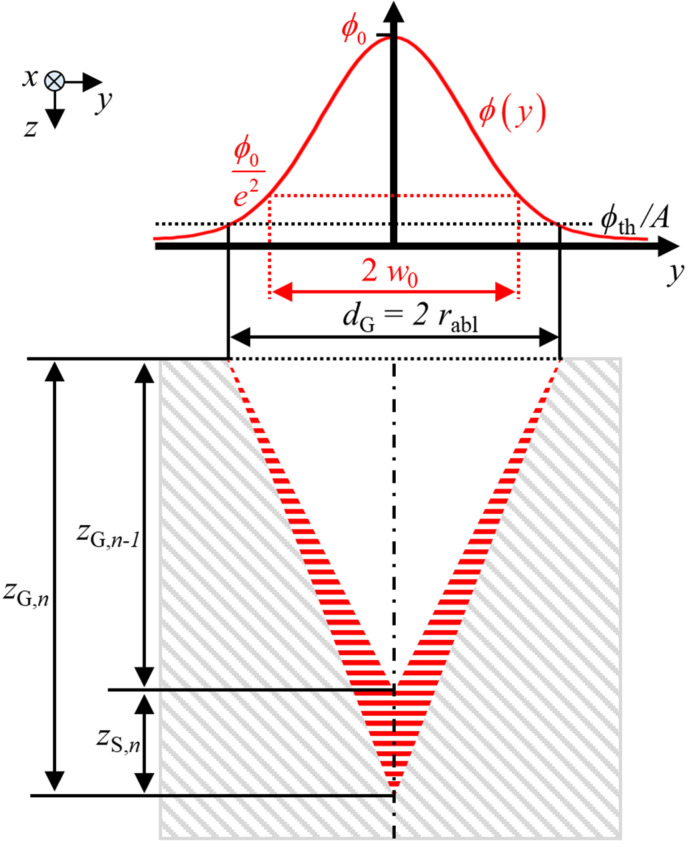
V-shaped groove produced by a pulsed laser beam which is scanned along the *x*-axis. The Gaussian distribution of the fluence of the individual laser pulses is shown by the red curve. The width of the groove is denoted by *d*_G_ = 2 *r*_abl_, which corresponds to two times the ablation radius *r*_abl_. The incrementally increased depth of the groove is denoted by *z*_G*,n*_, where *n* is the number of applied scans and *z*_S,*n*_ = *z*_G,*n*_ − *z*_G,*n* − 1_ is the incremental increase in the depth produced by the *n*th scan along the groove. The cross section of the volume ablated during the *n*th scan is highlighted by the red hatched cross section.

An analytical, recursive model for the calculation of the depth progress of laser-processed V-shaped grooves in metals can be derived in analogy to the model for percussion drilling of conical microholes as presented in [15]. Figure 1 illustrates the assumptions made for the model as seen by the cross section in the *y*–*z*-plane perpendicular to the scanning direction *x*. 

The pulses of a Gaussian laser beam are irradiated onto the metal sample (the grey hatched cross section) at normal incidence (i.e., in the *z*-direction). At the surface of the workpiece, the transversal distribution of the incident fluence (red curve) is given by

(1) $$\phi (x,y)=\phi_{0}\cdot \exp\left(-2\frac{{\left(x-x_{C}\right)}^{2}+{\left(y-y_{C}\right)}^{2}}{w_{0}^{2}}\right),$$

where *x − x_C_* and *y − y*_C_ are the distances from the centre of the laser beam located at (*x*_C_, *y*_C_), *w*_0_ is the beam radius and *ϕ*_0_ denotes the peak fluence, which is given by

(2) $$\phi_{0}=\frac{2\cdot E_{P}}{\pi \cdot w_{0}^{2}},$$

where *E*_P_ is the pulse energy. At normal incidence, material removal by ablation on the surface occurs when the locally absorbed fluence A⋅ϕ(y), where *A* is the material-specific absorptivity at the wavelength of the incident radiation, exceeds the value

(3) $$\phi_{\mathrm{th}}=l_{E}\cdot h_{V}$$

of the ablation threshold, where *l*_E_ denotes the effective penetration depth of the absorbed energy density and *h*_V_ denotes the volume-specific enthalpy required for heating and complete vaporization of the material. The effective penetration depth *l*_E_ is dominated either by the optical penetration depth or by the electron heat diffusion length, depending on the peak fluence of the incident radiation [22]. Additionally, *l*_E_ and thus *ϕ*_th_ decrease with increasing number of pulses applied to the surface [23] due to the so-called incubation effect [24]. The incubation effect saturates after about 100 pulses, whereupon the effective penetration depth and the ablation threshold are not significantly decreased further by additional pulses [23]. For the sake of simplicity, the energy penetration depth *l*_E_ and thus the ablation threshold *ϕ*_th_ are assumed to be constant over the entire process for the presented analytical model. The error caused by this simplification during the first 100 pulses is negligible as typically, more than several thousands of pulses are applied to each location for the production of laser machined grooves.

The width of the groove *d*_G_ resulting from material removal corresponds to two times the ablation radius *r*_abl_ and is calculated by [15]

(4) $$d_{G}=2\cdot r_{\mathrm{abl}}=2\cdot w_{0}\cdot \sqrt{\frac{1}{2}\cdot \ln\left(\frac{A\cdot \phi_{0}}{\phi_{\mathrm{th}}}\right)},$$

since no ablation can occur at the locations $\left|y\right|>r_{\mathrm{abl}}$ where the fluence absorbed on the surface of the workpiece is lower than the ablation threshold. It is implicitly premised here that the spatial overlap of consecutive pulses along the scan path in the *x*-direction is sufficiently large to ensure a constant width of the groove. The spatial pulse overlap Ω*_x_* is defined by

(5) $$\Omega_{x}=1-\frac{\delta_{x}}{2\cdot w_{0}},$$

where

(6) $$\delta_{x}=\frac{v_{x}}{f_{\mathrm{rep}}}$$

denotes the spatial offset between the impact locations of two consecutive pulses, *v_x_* is the scanning speed and *f*_rep_ is the pulse repetition rate. A constant groove width *d*_G_ is typically achieved with a spatial pulse overlap ranging from 30% to 95% [2]. 

The proposed recursive model is based on the assumption that the groove depth *z*_G,*n*_ after *n* ∈ 1,2,…*N* scans can be calculated by

(7) $$z_{G,n}=z_{G,n-1}+z_{S,n},$$

where *z*_G,*n −* 1_ denotes the groove depth after *n* − 1 scans and *z*_S,*n*_ denotes the depth ablated by the *n*th scan (cf. Figure 1). The overall absorptance *η_A_* resulting from multiple reflections inside the V-shaped groove may be calculated assuming specular reflections of a ray, which is incident in *z*-direction and is found to be [25]

(8) $$\eta_{A}\left(d_{G},z_{G,n-1}\right)=1-{\left(1-A\right)}^{N_{R}\left(d_{G},z_{G,n-1}\right)},$$

where *N*_R_ denotes the number of reflections of the ray until it leaves the groove again. This number of reflections depends on the aspect ratio of the V-shaped groove and is given by [25]

(9) $$N_{R}\left(d_{G},z_{G,n-1}\right)=\left\lceil \frac{\pi }{2\cdot \arctan\left(\frac{d_{G}}{2\cdot z_{G,n-1}}\right)}-\frac{1}{2}\right\rceil ,$$

where ⌈⌉ is the rounding up function. 

The energy *dE*_A,*n*_(*x_j_*) absorbed from one single pulse of a Gaussian laser beam during the *n*th scan along the groove at the location *x* in a stripe with the width *dx* inside the groove between the edges at *y* = ±*d*_G_/2 amounts to

(10) dEA,n,j(x)=dx⋅∫−dG2dG2ηA(dG,zG,n−1)⋅ϕ0⋅exp(−2⋅(x−xC,j)2+y2w02)dy,

where $x_{C,j}=j\cdot \delta_{x}$ is the location of the beam axis along the *x*-axis at the time at which the *j*th pulse hits the workpiece and where *j* ∈ ℤ and *y*_C_ was set to zero for the beam, which is centred on the groove. 

The overall absorptance *η_A_*(*d*_G_, *z*_G,*n* − 1_) only defines the amount of energy *dE*_A,*n,j*_(*x*) absorbed in the groove but does not specify the transversal distribution of the fluence in the *y*–*z*-plane (cf. Figure 1). As shown by raytracing simulations of V-shaped capillaries in [21], the effect of multiple reflections causes an elevated absorbed fluence near the tip of the groove. As a simple approximation for the transversal distribution of the absorbed fluence in the groove, it is assumed in the following that the absorbed fluence linearly increases with the depth along the sidewalls of the V-shaped groove in the *y*–*z*-plane. In analogy to the model presented for percussion drilling [15] and assuming that multiple reflections only occur normal to the axis of the groove, the distribution of the absorbed fluence at a given location *x* along the groove is assumed to start with *ϕ*_th_ at the edge of the groove (at *y* = ±*d*_G_/2) and end with *ϕ*_tip,*n*,*j*_(*x*) at the tip of the groove (*y* = *y*_C_ = 0). With this assumption, the energy *dE*_A,*n,j*_(*x*) absorbed at the location *x* from a single pulse *j* in a stripe of width *dx* amounts to

(11) $$dE_{A,n,j}\left(x\right)=dx\cdot \sqrt{d_{G}^{2}+4\cdot z_{G,n-1}^{2}}\cdot \left(\frac{\phi_{\mathrm{th}}+\phi_{\mathrm{tip},n,j}\left(x\right)}{2}\right),$$

where $\sqrt{d_{G}^{2}+4\cdot z_{G,n-1}^{2}}$ is the length of the two sidewalls together measured in the *y*–*z*-plane (cf. Figure 1). As both Equations (10) and (11) describe the same energy, it follows that

dx⋅dG2+4⋅zG,n−12⋅(ϕth+ϕtip,n,j(x)2)=dx⋅ηA(dG,zG,n−1)⋅ϕ0⋅exp(−2⋅(x−xC,j)2w02)⋅∫−dG2dG2exp(−2⋅y2w02)dy.

Inserting Equation (2) and replacing the integral with $\sqrt{\frac{\pi }{2}}\cdot w_{0}\cdot \mathrm{erf}\left(\frac{d_{G}}{\sqrt{2}\cdot w_{0}}\right)$, where erf is the well-known error function, one finds that the fluence deposited at the tip of the groove with the depth *z*_G,*n* − 1_ at the location *x* by the *j*th pulse during the *n*th scan is given by

(12) $$\phi_{\mathrm{tip},n,j}\left(x\right)=\frac{2\cdot \sqrt{2}\cdot \eta_{A}\left(d_{G},z_{G,n-1}\right)\cdot E_{P}}{\sqrt{\pi }\cdot w_{0}\cdot \sqrt{d_{G}^{2}+4\cdot z_{G,n-1}^{2}}}\cdot \mathrm{erf}\left(\frac{d_{G}}{\sqrt{2}\cdot w_{0}}\right)\cdot \exp\left(\frac{-2\cdot {\left(x-x_{C,j}\right)}^{2}}{w_{0}^{2}}\right)-\phi_{\mathrm{th}}.$$

**Figure 2:**
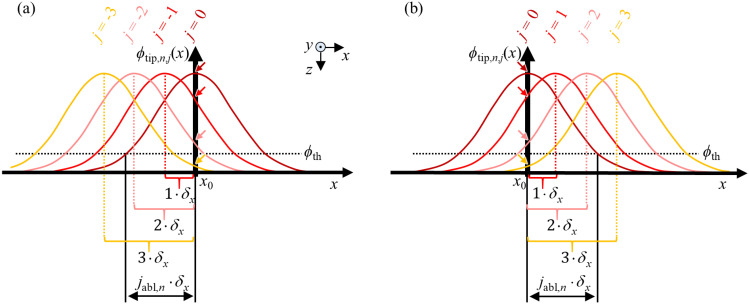
Absorbed fluence distributions *ϕ*_tip,*n*,*j*_(*x*) along the *x* axis at *y* = 0 of the incident pulses (**a**) from *j* = −3 to *j* = 0 and (**b**) from *j* = 0 to *j* = 3. The coloured small arrows indicate the absorbed fluence at the location *x*_0_. The intersection of the fluence distributions *ϕ*_tip,*n*,*j*_(*x*) with the ablation threshold *ϕ*_th_ determines the maximum number of pulses *j*_abl,*n*_ contributing to ablation in this direction along the *x* axis.

Figure 2 shows a sequence of distributions of the fluence *ϕ*_tip,*n*,*j*_(*x*) with a spatial offset of *δ_x_* each absorbed at the tip of the groove around an arbitrary point *x*_0_. For the sake of clarity, the figure is divided into two parts showing the pulses with *j* ≤ 0 in Figure 2a) and the pulses with *j* ≥ 0 in Figure 2b). The pulses are numbered in such a way that the beam axis coincides with *x*_0_ at the moment when the 0th pulse hits the workpiece (*x*_C,0_ = *x*_0_). Considering this diagram, it becomes evident that from the perspective of a point (*x* = *x*_0_, *y* = 0) located at *x*_0_ somewhere along the centre line of the groove, the individual pulses of a scan can only contribute to the ablation of the groove at this point *x*_0_ as long as the fluence absorbed at the tip *ϕ*_tip,*n*,*j*_(*x*_0_) > *ϕ*_th_ exceeds the ablation threshold *ϕ*_th_. The fluence that is absorbed at the tip of the groove from each of the pulses *j* of one scan (with *j* = …, −3, −2, −1, 0, 1, 2, 3, …) at the location *x* = *x*_0_ is given by the intersection of the fluence distribution *ϕ*_tip,*n*,*j*_(*x*) with the ordinate at *x* = *x*_0_, as indicated by the colored small arrows in Figure 2.

In the example depicted in Figure 2, only the pulses from *j* = −2 to *j* = 2 contribute to material ablation at (*x* = *x*_0_, *y* = 0), as only their fluences *ϕ*_tip,*n*,*j*_(*x*_0_) exceed the ablation threshold *ϕ*_th_, whose value is indicated by the black dotted line. The intersection $\phi_{\mathrm{tip},n,j}\left(x=j_{\mathrm{abl},n}\cdot \delta_{x}\right)=\phi_{\mathrm{th}}$ of the fluence distribution *ϕ*_tip,*n*,*j*_(*x*) with the ablation threshold *ϕ*_th_ determines the maximum number of pulses *j*_abl,*n*_ contributing to ablation in this direction. Using Equation (12) and solving for *j*_abl,*n*_ yields

(13) $$j_{\mathrm{abl},n}=\frac{w_{0}}{\delta_{x}}\cdot \sqrt{\frac{1}{2}\cdot \ln\left(\frac{\sqrt{2}\cdot \eta_{A}\left(d_{G},z_{G,n-1}\right)\cdot E_{P}\cdot \mathrm{erf}\left(\frac{d_{G}}{\sqrt{2}\cdot w_{0}}\right)}{\phi_{\mathrm{th}}\cdot \sqrt{\pi }\cdot w_{0}\cdot \sqrt{d_{G}^{2}+4\cdot z_{G,n-1}^{2}}}\right)}.$$

As a result, the depth ablated by the *n*th scan *z*_S,*n*_ as seen by the spot *x* = *x*_0_ located on the centre line of the groove corresponds to the accumulated depth ablated by the pulses $j=-\left\lfloor j_{\mathrm{abl},n}\right\rfloor$ to $j=\left\lfloor j_{\mathrm{abl},n}\right\rfloor$ and can be calculated by

(14) $$z_{S,n}=\sum_{j=-\left\lfloor j_{\mathrm{abl},n}\right\rfloor }^{\left\lfloor j_{\mathrm{abl},n}\right\rfloor }z_{P,n,j}\left(x\right),$$

where ⌊⌋ means rounding off and *z*_P,*n*,*j*_(*x*) denotes the depth ablated by the pulse *j* during the *n*th scan. According to the logarithmic ablation law [23,26], the depth increment ablated by a single pulse is given by

(15) $$z_{P,n,j}\left(x\right)=l_{E}\cdot \ln\left(\frac{\phi_{\mathrm{tip},n,j}\left(x\right)}{\phi_{\mathrm{th}}}\right).$$

In the present model, a constant absorptance *η_A_* (*d*_G_, *z*_G,*n* − 1_) as given by Equations (8) and (9) is assumed during one scan over the groove. This induces a negligible error since *η_A_* changes very slowly with an increasing number *n* of scans as long as *z*_S,*n*_ << *d*_G_, which is typically the case in micromachining processes with a reasonable pulse overlap Ω*_x_* in the range of 30–95%. 

With the above equations, the progress of the increasing groove depth *z*_G,*n*_ can be recursively calculated as a function of the number *n* of scans. A useful way to proceed is by starting with the calculation of the constant parameters that are not affected by the recursive calculation, such as the spatial offset *δ_x_* between the impact locations of two consecutive pulses using Equation (6). Furthermore, the peak fluence *ϕ*_0_ and ablation threshold *ϕ*_th_ can be calculated with Equations (2) and (3), respectively, in order to determine the width of the groove *d*_G_ using Equation (4). With the first scan (*n* = 1) at the beginning of the recursive calculation, a very small value should be chosen for the initial groove depth, e.g., *z*_G,0_ = 1 nm (*z*_G,0_ ≠ 0), so as not to divide by 0 in the subsequent calculation of the absorptance *η_A_* (*d*_G_, *z*_G,0_) in Equations (8) and (9). Then, the maximum number of pulses *j*_abl,1_ contributing to ablation in each direction is calculated using Equation (13), followed by the calculation of the fluence *ϕ*_tip,1,*j*_(*x*) deposited at the tip of the groove with Equation (12) for each contributing pulse *j* during this first scan. The depth increment *z*_P,1,*j*_(*x*) ablated by each pulse *j* is calculated using Equation (15) and accumulated according to Equation (14). Then, the accumulated depth of the first scan *z*_S,1_ is added to the initial groove depth *z*_G,0_ as given by Equation (7). The calculation of the absorptance *η_A_* (*d*_G_, *z*_G,1_) of the groove with increased depth *z*_G,1_ starts the second loop of the recursive calculation. This procedure must be repeated *n* times to receive the groove depth *z*_G,*n*_ after micromachining with *n* scans.

The absorbed fluence *ϕ*_tip,*n*,*j*_(*x*) at the tip of the groove decreases with increasing groove depth due to the increasing length of the sidewalls $\sqrt{d_{G}^{2}+4\cdot z_{G,n-1}^{2}}$. This reduction is partially compensated by an increased absorptance *η_A_* (*d*_G_, *z*_G,*n*−1_) due to the increasing number *N*_R_ of reflections within the groove (cf. Equations (8) and (9)). The maximum attainable groove depth *z*_G*,*∞_ obtained after *n* → ∞ scans is reached when the fluence *ϕ*_tip,*n*,*j*_(*x* = *x*_C_) at the tip of the V-shaped groove converges to the value of the ablation threshold *ϕ*_th_. The maximum groove depth *z*_G*,*∞_ can therefore be found with Equation (12) by setting *ϕ*_tip,*n*,*j*_(*x* = *x*_C_) = *ϕ*_th_, and solving for *z*_G*,*∞_, which yields

(16) $$z_{G,\infty }=\sqrt{\frac{\eta_{\infty }{}^{2}\cdot E_{P}^{2}}{2\cdot \pi \cdot w_{0}^{2}\cdot \phi_{\mathrm{th}}^{2}}\cdot {\left(\mathrm{erf}\left(\frac{d_{G}}{\sqrt{2}\cdot w_{0}}\right)\right)}^{2}-\frac{d_{G}^{2}}{4}},$$

where *η*_∞_ = *η_A_* (*d*_G_, *z*_G,*∞*−1_) denotes the absorptance of a groove micromachined with ∞−1 scans. As the absorptance, in turn, depends on the groove depth *z*_G,*n*−1_ (cf. Equations (8) and (9)), the maximum groove depth *z*_G*,∞*_ cannot be calculated directly but has to be found by a recursive calculation using Equation (7). Assuming a high aspect ratio *z*_G,∞_/*d*_G_ of the final groove, the absorptance can, however, be approximated to be *η*_∞_ ≈ 1, and the maximum achievable groove depth *z*_G,∞_ obtained with a given parameter set can directly be estimated using Equation (16) by setting *η_∞_* = 1. Equation (16) also shows that the maximum achievable groove depth does neither depend on the repetition rate *f*_rep_ nor on the scanning parameters such as the scanning speed *v_x_* and that—for a given beam radius *w*_0_ and with the material-specific value of *ϕ*_th_—it can only by increased by increasing the pulse energy *E*_P_.

It is noted that the model for the calculation of the depth progress presented in this section only requires five generally known laser and scanning parameters, namely the pulse energy *E*_P_, the repetition rate *f*_rep_, the radius *w*_0_ of the laser beam, the scanning speed *v_x_*, and the number of scans *n*, as well as the three material parameters, absorptivity *A*, energy penetration depth *l*_E_, and the enthalpy *h*_V_ for heating and complete vaporization of the material.

## 3. Experimental Verification of the Analytical Model

The model for the prediction of the depth and width of laser micromachined grooves derived in the previous section was compared to experimental results obtained by micromachining samples with a size of 50 × 50 mm² and a thickness of 1 mm made of Ti6Al4V (ASTM Grade 5), a Ti-alloy often used in the aerospace industry and for biomechanical applications due to the high specific strength, corrosion resistance and biocompatibility. The ultrafast laser system *Pharos* from *Light Conversion* with a wavelength of 1030 nm was used for micromachining. The laser emitted pulses with a pulse duration of 260 fs. The circularly polarized laser beam with a Gaussian intensity distribution had a beam propagation factor of M² < 1.3. The beam was scanned over the surface of the samples by means of a Galvanometer-Scanner (*Scanlab*, *intelliSCAN* 30) and was focused by an F-Theta lens (*Sill Optics*, *S4LFT*1330/328) with a focal length of 340 mm, resulting in a focal radius of *w*_0_ = 55 ± 5 µm. The focus position was always set on the surface of the samples. Grooves with a length of 10–35 mm were micromachined in the Ti-samples with different pulse energies *E*_P_, repetition rates *f*_rep_, scanning speeds *v_x_* and number of scans *n,* as summarized in Table 1. The spatial offset *δ_x_* of the impact locations of two consecutive pulses and the corresponding pulse overlap Ω*_x_* were calculated according to Equation (6) and Equation (5), respectively.

**Figure 3:**
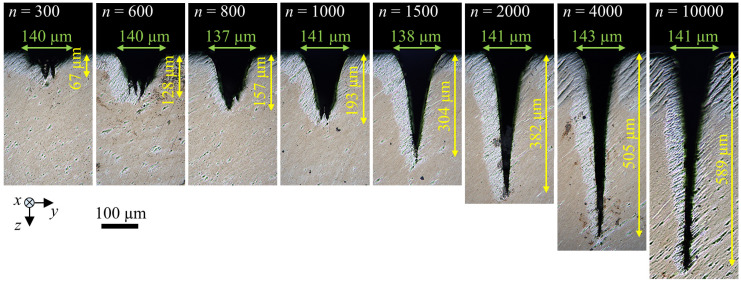
Cross sections of grooves micromachined in Ti6Al4V with the parameter set P1. The depth and width of each groove are indicated by a yellow double arrow and a green double arrow, respectively.

After micromachining, the samples were cut perpendicular to the grooves (*y*–*z*-plane), and cross sections were prepared by grinding and polishing in order to investigate the shape of the grooves and measure their depth and width using an optical microscope (Leica, DM6 M). Figure 3 shows the cross sections obtained with the parameter set P1 (cf. Table 1). 

Five grooves were machined with each of the parameter sets listed in Table 1. The measured values of the depth and width of the five grooves from each parameter set were used for averaging and calculating the maximum and minimum deviation. As expected from theory, the width of the groove remained constant at the value of *d*_G_ = $138_{-5}^{+8}$ μm independent of the number of scans, which is also in good agreement with the results shown in [2]. The V-shape clearly dominates the shape of the shown grooves for *n* ≥ 800. Deviations from the V-shape can be seen for *n* = 300 and *n* = 600 due to rough structures at the bottom of the grooves. Bending of the tip of the groove occurred for *n* = 10,000, which was also observed in [8] for micromachining of deep grooves in a Ni-alloy and drilling of deep microholes in CVD diamond [27]. The cause for the bending of the tip has not been conclusively clarified yet, but a polarization-dependent behaviour was found in [27].

The material parameters for titanium published in [28,29] were used for the calculation of the volume-specific enthalpy of *h*_V_ = 47.1 J/mm³, which is required to heat and vaporize the material. The values are listed in Table 2. The absorptivity of titanium at normal incidence and at a wavelength of 1030 nm was set to *A* = 0.51 [30]. The effective penetration depth was used as a fit parameter. A good agreement between the calculated and the experimental results was found with *l*_E_ = 30 nm. This value corresponds to an absorbed threshold fluence of *ϕ*_th_ = 0.14 J/cm² (cf. Equation (3)). The fitted value of *l*_E_ = 30 nm is consistent with experimentally determined values of the optical penetration depth of 26 nm for Ti6Al4V [31] and 30 nm for titanium [22].

The groove widths are given by Equation (4) for the peak fluences of 3.81 J/cm² (P1) and 1.45 J/cm² (P2) yield *d*_G_ = 126 µm and *d*_G_ = 100 µm, respectively. The experimentally determined widths of $138_{-5}^{+8}$ µm (P1) and $113_{-5}^{+4}$ µm (P2) are slightly larger. The moderate deviations of less than 15% may be explained by the fact that no incubation effect is taken into account in the model.

**Figure 4:**
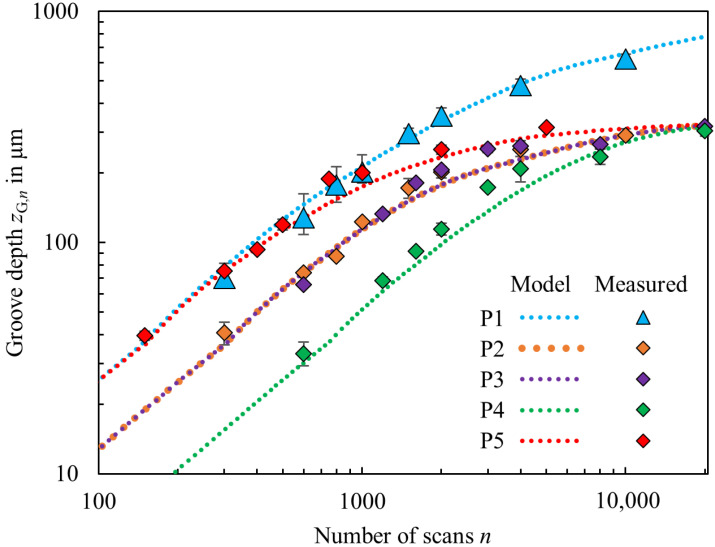
Calculated groove depth (dotted lines, “Model”) and measured groove depth (data points, “Measured”) as a function of the number of scans for grooves micromachined in Ti6Al4V using the different parameter sets as given in Table 1.

The progress of the groove depth *z*_G,__*n*_ as a function of the number *n* of scans was recursively calculated as described above. The calculations are compared to the experimental results in Figure 4. The groove depths as calculated by the model derived in the previous section and as measured from the cross sections for the different parameter combinations P1–P5 (cf. Table 1) are represented in different colors with dotted lines and data points, respectively. The value of the data points corresponds to the average values measured from up to five grooves micromachined with identical parameters. The error bars represent the deviation to the maximum and minimum measured groove depth of each parameter set.

Up to an aspect ratio (depth/width) of *z*_G_/*d*_G_ ≈ 1.5, the measured groove depth increases almost linearly with the number of scans. The progress of the depth is found to slow down for aspect ratios beyond *z*_G_/*d*_G_ > 1.5. At constant repetition rate *f*_rep_ and scanning speed *v_x_*, higher depth progress and deeper grooves were achieved for higher pulse energies (cf. P1 and P2). For constant pulse energy *E*_P_ and constant pulse overlap Ω*_x_*, the groove depth as a function of number of scans is similar (cf. P2 and P3). However, the net processing time is divided in half for P3 in comparison to P2 due to double the scanning speed *v*_x_ at a twofold repetition rate *f*_rep_. At constant pulse energy *E*_P_ and constant repetition rate *f*_rep_, higher depth progress is achieved with lower scanning speeds *v_x_* (cf. P3, P4 and P5). The relations observed in this work regarding the depth progress in micromachining of grooves in Ti6Al4V confirm the observations made for semiconductors in [2,9] and for a Ni-alloy in [8]: The groove depth increases with increasing number of scans, and at high pulse energies and low scanning speeds, a greater increase in depth was observed with each scan. The maximum groove depth of $624_{-38}^{+28}$ µm was achieved with the highest investigated pulse energy *E*_P_ = 181 µJ and the highest number of scans *n* = 10,000 for this parameter combination (P1). The maximum measured groove depth for a constant pulse energy *E*_P_ = 69 µJ and different scanning parameters (from P2 to P5) is in the range of $306_{-14}^{+13}$  µm.

The groove depths as calculated by the model (dotted lines, “Model”) and as measured by the cross sections with the optical microscope (data points, “Measured”) are in very good agreement for the different parameter combinations and for the different number of scans. The depth progress predicted by the model decreases with increasing number of scans and stagnates when the fluence in the tip converges the ablation threshold, which corresponds well with the results from [2]. As a result, the calculated groove depths as a function of the number of scans for a constant pulse energy *E*_P_ = 69 µJ, but micromachined with different scanning parameters (from P2 to P5) converge to the same maximum groove depth, which agrees well with the theoretical prediction of the model of 326 µm calculated by Equation (16) for *η_∞_* = 1. Deviations from calculation and measurement might result from uncertainties regarding the material parameters used for the calculation, in particular the fitted value for the effective penetration depth *l*_E_, or from deviations of the assumed ideal V-shape, as shown before in Figure 3 for *n* = 10,000, with the bending of the tip. Complete vaporization is assumed in the proposed analytical model, whereas additional effects such as melting and spallation can cause a deviating process enthalpy and thus a different ablation rate [32].

**Figure 5:**
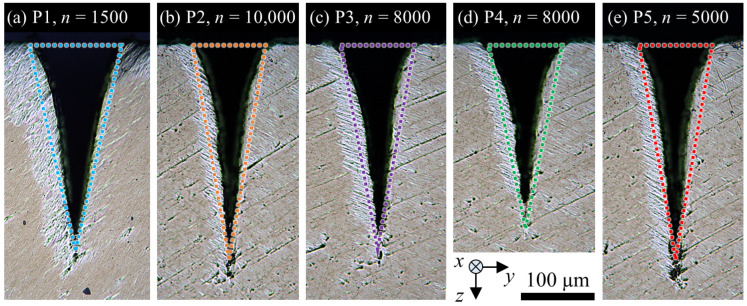
Cross sections of grooves micromachined in Ti6Al4V with (**a**) P1, *n* = 1500, (**b**) P2, *n* = 10,000, (**c**) P3, *n* = 8000, (**d**) P4, *n* = 8000, and (**e**) P5, *n* = 5000. An isosceles triangle with the dimensions of the calculated depth and width of the corresponding groove using the model presented in Section 2 is inserted for each parameter set in the respective colour.

Nevertheless, for a broad range of laser and scanning parameters, the V-shape and calculated groove dimensions by the model correspond to the shape and groove dimensions as measured by the cross sections shown in Figure 5 for some of the grooves from P1 to P5 and different number of scans. The colored triangles were dimensioned according to the groove depth *z*_G_ and groove width *d*_G_, as calculated by the model derived in the previous section.

Knowing the laser parameters *E*_P_ and *f*_rep_, the scanning speed *v_x_* and beam radius *w*_0_, and the three material parameters *A*, *l*_E_ and *h*_V_, the model allows for the prediction of the groove dimensions as a function of the number of scans *n* and maximum achievable groove depth.

## 4. Conclusions

An analytical model for the prediction of the depth and width of V-shaped grooves in metals micromachined with ultrashort laser pulses was derived. The model predicts the progress of the micromachining depth for V-shaped grooves in a Ti-alloy with ultrashort laser pulses as a function of laser parameters, scanning parameters and material parameters. The corresponding assumptions for the model were experimentally validated for different pulse energies, repetition rates, scanning speeds and number of scans using cross sections and optical microscopy. The analytical model derived in our paper provides a useful tool for the estimation of the groove dimensions, process windows of micromachining with high depth progress and the maximum achievable groove depth.

## Figures and Tables

**Table 1:** Sets of parameters as used for micromachining of grooves with different depths and widths.

	*P*_av_ in W	*E*_P_ in µJ	*ϕ*_0_ in J/cm²	*f*_rep_ in kHz	*v_x_* in m/s	*δ_x_* in µm	Ω*_x_*	Number of Scans *n*
P1	9.05	181	3.81	50	1.2	24	78%	300…10,000
P2	3.45	69	1.45	50	1.2	24	78%	300…10,000
P3	6.90	69	1.45	100	2.4	24	78%	600…20,000
P4	3.45	69	1.45	50	2.4	48	56%	600…20,000
P5	3.45	69	1.45	50	0.6	12	89%	150…5000

**Table 2:** Material properties of titanium, as published in [28,29] used for the calculation of the volume-specific enthalpy *h*_V_ for heating and vaporization.

Material Parameter	Value
Density	$4506\frac{\mathrm{kg}}{m^{3}}$ [28]
Heat capacity for solid titanium	$523\frac{J}{\mathrm{kg}\cdot K}$ [28]
Melting temperature	1668 °C [28]
Latent heat of melting	$440\frac{\mathrm{kJ}}{\mathrm{kg}}$ [29]
Vaporization temperature	3287 °C [28]
Latent heat of vaporization	$8305\frac{\mathrm{kJ}}{\mathrm{kg}}$ [29]

## Data Availability

The data presented in this study are available on request from the corresponding author.
